# PHYSICAL FUNCTIONING AND BODY SIZE IN MID-LIFE WOMEN: THE STUDY OF WOMEN’S HEALTH ACROSS THE NATION

**DOI:** 10.1093/geroni/igac059.2462

**Published:** 2022-12-20

**Authors:** Minsuk Oh, Kelly Ylitalo, Aleda Leis, Brittney Lange-Maia, Elsa Strotmeyer, Jane Cauley, Carrie Karvonen-Gutierriez

**Affiliations:** Baylor University, Woodway, Texas, United States; Baylor University, Waco, Texas, United States; University of Michigan, Ann Arbor, Michigan, United States; Rush University, Chicago, Illinois, United States; School of Public Health, University of Pittsburgh, Pittsburgh, Pennsylvania, United States; University of Pittsburgh, Pittsburgh, Pennsylvania, United States; University of Michigan, Ann Arbor, Michigan, United States

## Abstract

To evaluate patterns of age-related changes in physical functioning (PF) and associations with body size, we utilized data from the longitudinal Study of Women’s Health Across the Nation (SWAN). Participants (n=1,793) with self-reported SF-36 PF data at visit 4 (2000-01; mean age:50.0 years ±2.7), visit 15 (2016-17), plus one additional visit were included. Body weight and waist circumference were measured at each visit; change from visits 4 to 15 was calculated. Five PF trajectories were identified using latent class growth modeling (% of women): (1) persistently low (4.1%); (2) start moderate and improve slightly (5.4%); (3) start high and decline slightly (24.0%); (4) persistently high (59.5%); and (5) start high and decline substantially (7.0%). Participants with persistently low PF (Group 1) lost weight during follow-up (mean change: -3.2% body weight) whereas there was little change in all other groups (ANOVA p< 0.0001). Women with persistently low PF (Group 1) had < 2% increase in waist circumference over follow-up; all other groups had more than double that increase (range: 4.1%-6.1%; ANOVA p=0.002). No statistically significant differences in anthropometry changes across the PF groups were observed after adjustment for sociodemographic and time-variant health characteristics. PF trajectories may be associated with changes in body weight or waist circumference during midlife and transition to older adulthood; however, these associations may be explained by other major health-related variables. Future research is needed to understand the complex interplay between PF and body size, particularly for women at-risk for late-life disability that may benefit from preventive efforts in mid-life.

